# The Association between Daytime Napping Characteristics and Bone Mineral Density in Elderly Thai Women without Osteoporosis

**DOI:** 10.1038/s41598-018-28260-w

**Published:** 2018-07-03

**Authors:** Sunee Saetung, Sirimon Reutrakul, La-or Chailurkit, Rajata Rajatanavin, Boonsong Ongphiphadhanakul, Hataikarn Nimitphong

**Affiliations:** 1 Division of Endocrinology and Metabolism, Department of Medicine, Faculty of Medicine, Ramathibodi Hospital, Mahidol University, Bangkok, 10400 Thailand; 2 Division of Endocrinology, Diabetes and Metabolism, University of Illinois at Chicago, Chicago, IL USA

## Abstract

Low bone mass is more prevalent with increasing age. Studies have found associations between sleep duration, sleep quality and obstructive sleep apnea and bone mineral density (BMD). However, less is known about the relationship between daytime napping and BMD. We aimed to investigate the association between daytime napping and BMD in elderly Thai women. Demographic data, lifestyle information and sleep characteristics were obtained by interviewing 387 elderly women. Weight and height were measured. Serum 25-hydroxyvitamin D [25(OH)D] was measured by radioimmunoassay. BMD was measured by dual-energy X-ray absorptiometry (DXA). Higher BMI and having type 2 diabetes (T2DM) were correlated with higher lumbar spine 2–4 (L2-4) BMD, while younger age, higher BMI and higher serum 25(OH)D level were correlated with higher femoral neck (FN) and total hip (TH) BMD. After adjusting for age, age at menopause, BMI, 25(OH)D level and T2DM, a higher frequency of weekly daytime napping was associated with lower FN and TH BMD but not at L2-4 BMD. Additionally, longer daytime napping duration was negatively associated with BMD at TH. In summary higher frequency and longer duration of daytime napping are associated with lower femoral BMD in elderly women. Mechanisms underlying these associations should be further explored.

## Introduction

Low bone mass and osteoporosis are major public health problems worldwide. Genetics, sex, age, hormonal status, lifestyle factors such as calcium intake, exercise, smoking and alcohol consumption are well-known factors affecting bone health^1,2^. Recently, increasing evidence has suggested that sleep characteristics, including sleep apnea and nighttime and daytime sleep duration, are related to osteoporosis. Some studies have demonstrated an association between more severe sleep apnea and increased risk of osteoporosis^3,4^. Others have examined the relationship between nighttime sleep duration and bone health. Postmenopausal women and elderly participants, but not younger individuals, with either short or long nighttime sleep duration were found to have low bone mineral density (BMD)^5–7^. The relationship between sleep deprivation and bone markers and BMD was explored in an experimental sleep restriction protocol. In 10 healthy men, after three weeks of sleep restriction (5.6 h sleep/24 h)^8^, levels of the bone formation marker N-terminal propeptide of type 1 procollagen (P1NP) significantly decreased from baseline, while the bone resorption marker C-terminal cross-linked telopeptide of type I collagen (CTX) did not change.

Besides sleep duration and sleep apnea, daytime napping is another sleep characteristic which should be considered. Daytime napping is common in the elderly. Only a few studies have explored the association between daytime napping and BMD^5,9^. In one study, daytime napping duration ≥30 min in postmenopausal women (n = 4,962) and ≥60 min in premenopausal women (n = 1,548) were found to be associated with osteopenia and osteoporosis, as diagnosed by calcaneal qualitative ultrasound (QUS)^9^. However, the mechanisms underlying this relationship have not been established.

Collectively, osteoporosis and changes in sleep patterns are two common findings in the elderly. However, the link between daytime napping and BMD in the elderly has not been well explored. The objective of this study was to investigate the association between sleep patterns, including nighttime sleep duration and daytime napping, and BMD in elderly Thai women.

## Results

Three hundred and eighty-seven elderly Thai women with mean age of 66.2 years and mean age at menopause of 49.4 years were included in this study. Baseline demographics, BMD and sleep characteristics are shown in Table 1. Mean BMI was 25.4 kg/m^2^. Mean dietary calcium intake was 371.6 mg/day and mean serum 25(OH)D was 68.2 nmol/L. About 16% of the subjects had 25(OH)D of less than 50 nmol/L, and 15.5% of the subjects had a diagnosis of type 2 diabetes (T2DM). Only 12 (3.1%) and 4 (1%) of the subjects had calcium and vitamin D supplementation prior to two months before the enrollment, respectively. In addition, 1.3% and 2.1% of subjects were current smokers and regular alcohol users, respectively. One-half of the subjects reported regular exercise. Fifteen subjects (3.9%) reported previous osteoporotic fractures and all of those were wrist fractures. Mean (SD) of L2-4, FN and TH BMD were 1.008 (0.132), 0.780 (0.101) and 0.877 (0.107) g/cm^2^, respectively. According to BMD *T*-score, 68.7% of subjects had osteopenia. For sleep characteristics, mean nighttime sleep duration was 6.9 h (range 3.0 to 10.0 h), and nearly three-fourths (71.3%) reported daytime napping. Overall, mean weekly daytime napping frequency and duration were 3.4 times/week and 3.4 h/week, respectively.

**Table 1:** Baseline demographics, bone mineral density and sleep characteristics.

Variables	Mean (SD) *N* = 387	Range
Age (years)	66.2 (4.3)	60.0–85.1
Age at menopause (years)^a^	49.4 (4.9)	28.0–60.0
Weight (kg)	59.4 (8.5)	37.6–84.9
Height (cm)	153.0 (5.0)	136.2–167.5
BMI (kg/m^2^)	25.4 (3.5)	16.5–36.3
Dietary calcium intake (mg/day)	371.6 (203.9)	52.9–1,491.2
Serum 25(OH)D (nmol/L)^b^	68.2 (16.0)	15.2–136.6
Serum 25(OH)D (nmol/L)^b^		
<50 nmol/L (*n*) (%)	62 (16.2)	
≥50 nmol/L (*n*) (%)	321(83.8)	
Previous calcium supplementation (n) (%)	12 (3.1)	
Previous vitamin D supplementation (*n*) (%)	4 (1)	
Diabetes		
No (*n*) (%)	327 (84.5)	
Yes (*n*) (%)	60 (15.5)	
Current smoker		
No (*n*) (%)	382 (98.7)	
Yes (*n*) (%)	5 (1.3)	
Regular alcohol consumption		
No (*n*) (%)	379 (97.9)	
Yes (*n*) (%)	8 (2.1)	
Exercise		
No (*n*) (%)	190 (49.1)	
Yes (*n*) (%)	197 (50.9)	
Previous osteoporotic fractures		
No (*n*) (%)	372 (96.1)	
Yes (*n*) (%)^c^	15 (3.9)	
**Bone mineral density**		
Lumbar spine 2–4 (g/cm^2^)	1.008 (0.132)	0.826–1.544
Femoral neck (g/cm^2^)	0.780 (0.101)	0.610–1.142
Total hip (g/cm^2^)	0.877 (0.107)	0.641–1.213
Normal BMD (*T*-score >−1) (*n*) (%)	121 (31.3)	
Osteopenia (*T*-score ≤ −1 and > −2.5) (*n*) (%)	266 (68.7)	
**Sleep characteristics**		
Nighttime sleep duration (h)	6.9 (1.2)	3.0–10.0
Reported daytime napping		
No (*n*) (%)	111 (28.7)	
Yes (*n*) (%)	276 (71.3)	
Daytime napping frequency per week	3.4 (2.9)	0–7
Daytime napping duration per week (h)	3.4 (4.1)	0–28

BMI, body mass index; 25(OH)D, 25-hydroxyvitamin D; BMD, bone mineral density; ^a^n = 386; ^b^n = 383; ^c^all of osteoporotic fractures were wrist fractures. Data are expressed as mean (SD) and range or percentage. SD, standard deviation.

### Associations between L2-4, FN and TH BMD and Demographic Variables, Lifestyle Variables and Sleep Characteristics

Univariate regression analyses were performed to examine the associations between L2-4, FN and TH BMD and demographic variables, lifestyle variables and sleep characteristics (Table 2). For associations with L2-4 BMD: higher BMI (unstandardized coefficient, *B* = 0.008, *p* < 0.001) and having T2DM (*B* = 0.039, *p* = 0.034) were associated with higher L2-4 BMD. There was a trend of positive association between age at menopause and L2-4 BMD (*B* = 0.003, *p* = 0.060). Age, dietary calcium intake, 25(OH)D level, calcium supplementation, vitamin D supplementation, exercise, previous osteoporotic fractures and sleep characteristics were not correlated with L2-4 BMD.

**Table 2:** Univariate linear regression analyses between lumbar spine L2-4, femoral neck and total hip BMD and participants’ characteristics.

Variables	Lumbar spine L2-4		Femoral neck		Total hip	
	*B*	*p*-value	*B*	*p*-value	*B*	*p*-value
Age (years)	−0.001	0.424	−0.007	<***0.001***	−0.006	<***0.001***
Age at menopause (years)^a^	0.003	***0.060***	0.002	***0.091***	0.001	0.213
BMI (kg/m^2^)	0.008	<***0.001***	0.005	***0.001***	0.008	<***0.001***
Dietary calcium intake (mg/day)	−0.00003	0.255	0.00003	0.219	0.00002	0.479
Serum 25(OH)D (nmol/L)^b^	0.001	0.234	0.001	***0.027***	0.001	***0.017***
Previous calcium supplementation	0.017	0.670	0.037	0.218	0.001	0.974
Previous vitamin D supplementation	−0.028	0.400	−0.013	0.611	−0.009	0.746
Diabetes	0.039	***0.034***	−0.009	0.540	0.015	0.316
Exercise	0.013	0.344	0.015	0.135	0.016	0.151
Previous osteoporotic fractures^c^	−0.017	0.135	−0.011	0.217	−0.010	0.283
Nighttime sleep duration (h)	−0.003	0.560	−0.001	0.872	0.000	0.954
Reported daytime napping	−0.013	0.397	−0.001	0.929	−0.008	0.501
Daytime napping duration per week (h)	0.000	0.801	−0.001	0.413	−0.012	0.188
Daytime napping frequency per week	0.000	0.881	−0.003	0.141	−0.003	***0.089***

BMI, body mass index; 25(OH)D, 25-hydroxyvitamin D = 386; ^b^n = 383; ^c^all of osteoporotic fractures were wrist fractures. Bolded values indicate statistical significance at p < 0.1.

For associations with FN BMD: younger age (*B* = −0.007, *p* < 0.001), higher BMI (*B* = 0.005, *p* = 0.001) and higher 25(OH)D level (*B* = 0.002, *p* = 0.027) were associated with higher FN BMD. Dietary calcium intake, calcium supplementation, vitamin D supplementation, T2DM, exercise, previous osteoporotic fractures and sleep characteristics were not correlated with FN BMD.

For associations with TH BMD: younger age (*B* = −0.006, *p* < 0.001), higher BMI (*B* = 0.008, *p* < 0.001) and higher 25(OH)D level (*B* = 0.002, *p* = 0.017) were associated with higher TH BMD. Similar to FN BMD, there were no associations between TH BMD and calcium intake, calcium supplementation, vitamin D supplementation, T2DM, exercise, previous osteoporotic fractures and sleep characteristics. However, higher weekly napping frequency tended to be associated with higher TH BMD (*B* = −0.003, *p* = 0.089).

### A Hierarchical Regression Analysis with L2-4 BMD, FN and TH BMD as Outcomes

We further investigated whether daytime napping could predict the variance in BMD, in addition to the relevant variables, using a hierarchical regression analysis. In this analysis, variables associated with L2-4, FN and TH BMD – including age, age at menopause, BMI, serum 25(OH)D level and a diagnosis of T2DM were entered in the first step. In the second step, napping characteristics (weekly frequency or weekly duration) were entered. Table 3 demonstrates the hierarchical multiple linear regression analysis between predictor variables (demographic data and daytime napping frequency/duration) and L2-4 BMD. Age, age at menopause, BMI, serum 25(OH)D level and DM explained 7.9% of the variance in L2-4 BMD. Both weekly daytime napping frequency and duration did not additionally explain the variance in L2-4 BMD (Table 3).

**Table 3:** Hierarchical multiple linear regression with lumbar spine L2-4 BMD as an outcome (n = 382).

Variables	Model 1		Model 2		Model 3	
	*B*	*p*-value	*B*	*p*-value	*B*	*p*-value
Age	−0.002	0.235	−0.002	0.297	−0.002	0.237
Age at menopause (years)	0.004	***0.008***	0.004	***0.008***	0.004	***0.007***
BMI (kg/m^2^)	0.009	**<** ***0.001***	0.010	**<** ***0.001***	0.010	**<** ***0.001***
Serum 25(OH)D (nmol/L)	0.001	0.098	0.001	0.091	0.001	0.122
Diabetes	0.041	***0.025***	0.043	***0.039***	0.041	***0.025***
Daytime napping frequency/week			−0.003	0.202		
Daytime napping duration/week (h)					−0.001	0.361
Adjusted *R*^2^	0.079		0.081		0.079	
∆*R*^2^			0.004	0.202	0.002	0.361

BMI, body mass index; BMD, bone mineral density; 25(OH)D, 25-hydroxyvitamin D. Bolded values indicate statistical significance at p < 0.05.

At FN BMD: age, age at menopause, BMI, serum 25(OH)D level and a diagnosis of DM explained 12.4% of the variance in FN BMD (Table 4). Weekly daytime napping frequency was added in the second step. This revealed that higher weekly daytime napping frequency was significantly associated with lower FN BMD (*B* = −0.004, *p* = 0.017). The second model explained an additional 1.3% of the variance in FN BMD (∆*R*^2^ = 0.013, *p* = 0.017, total adjusted *R*^2^ = 0.135). However, adding weekly daytime napping duration in the third model did not additionally explain the variance in FN BMD.

**Table 4:** Hierarchical multiple linear regression with femoral neck BMD as an outcome (n = 382).

Variables	Model 1		Model 2		Model 3	
	*B*	*p*-value	*B*	*p*-value	*B*	*p*-value
Age (years)	−0.007	<***0.001***	−0.007	<***0.001***	−0.007	<***0.001***
Age at menopause (years)	0.002	***0.020***	0.003	***0.010***	0.002	***0.015***
BMI (kg/m^2^)	0.005	<***0.001***	0.006	<***0.001***	0.005	<***0.001***
Serum 25(OH)D (nmol/L)	0.001	***0.006***	0.001	***0.008***	0.001	***0.009***
Diabetes	0.002	0.891	0.005	0.739	0.002	0.891
Daytime napping frequency/week			−0.004	***0.017***		
Daytime napping duration/week (h)					−0.001	0.236
Adjusted *R*^2^	0.124		0.135		0.125	
∆*R*^2^			0.013	***0.017***	0.003	0.236

BMI, body mass index; BMD, bone mineral density; 25(OH)D, 25-hydroxyvitamin D. Bolded values indicate statistical significance at p < 0.05.

At TH BMD: age, age at menopause, BMI, serum 25(OH)D level and a diagnosis of DM explained 15% of the variance in total hip BMD (Table 5). Daytime napping frequency was added in the second step. There was a significant association between higher daytime napping frequency and lower TH BMD (*B* = −0.005, *p* = 0.003), independent of relevant variables. This model explained an additional 2.0% of the variance in TH BMD (∆*R*^2^ = 0.020, *p* = 0.003, total adjusted *R*^2^ = 0.168). In addition, when daytime napping duration was added in the third model, it explained an additional 0.9% of the variance in TH BMD (∆*R*^2^ = 0.009, *p* = 0.043, total adjusted *R*^2^ = 0.157).

**Table 5:** Hierarchical multiple linear regression with total hip BMD as an outcome (n = 382).

Variables	Model 1		Model 2		Model 3	
	*B*	*p*-value	*B*	*p*-value	*B*	*p*-value
Age (years)	−0.006	<***0.001***	−0.006	<***0.001***	−0.006	<***0.001***
Age at menopause	0.002	***0.037***	0.003	***0.015***	0.002	***0.023***
BMI (kg/m^2^)	0.009	<***0.001***	0.009	<***0.001***	0.009	<***0.001***
Serum 25(OH)D (nmol/L)	0.001	***0.002***	0.001	***0.003***	0.001	***0.004***
Diabetes	0.022	0.123	0.026	0.072	0.022	0.121
Daytime napping frequency/week			−0.005	***0.003***		
Daytime napping duration/week (h)					−0.003	***0.043***
Adjusted *R*^2^	0.150		0.168		0.157	
∆*R*^2^			0.020	***0.003***	0.009	***0.043***

BMI, body mass index; BMD, bone mineral density; 25(OH)D, 25-hydroxyvitamin D. Bolded values indicate statistical significance at p < 0.05.

In addition, the associations between daytime napping and BMD at all sites were reanalyzed using stepwise regression analysis. All of the results were similar (data not shown).

## Discussion

This study demonstrated that higher weekly napping frequency was independently associated with lower FN and TH BMD after adjusting for known factors related to BMD. Moreover, longer weekly napping duration was associated with lower TH BMD. Beyond the traditional predictors for BMD, an increase in napping frequency of once per week predicted additional variance in FN and TH BMD of 1.3 and 2.0%, respectively, and an increase in weekly daytime napping duration of 1 h predicted additional variance in TH BMD of 0.9%. Subjects who napped 7 times/week had 0.028 g/cm^2^ (3.6%) lower FN BMD and 0.035 g/cm^2^ (4%) lower TH BMD than subjects who did not nap. These differences are close to the least significant change in hip BMD (≥4% in general)^10^ and are considered clinically significant. Further studies should explore whether modifications of napping would be beneficial for bone health, along with possible underlying mechanisms.

Napping frequency may represent a novel risk factor for low BMD, possibly leading to osteoporotic fracture. Only one study previously assessed the association between napping frequency and fractures, although BMD data were not available^11^. In this study of 8,101 Caucasian women (mean age 69), those who reported daily napping were 33% more likely to suffer a hip fracture than women who did not nap daily, after a median follow-up of 6.8 years^11^. Our data further supported these findings by demonstrating the detrimental effect of frequent napping on FN and TH BMD.

The association between longer weekly napping duration and lower BMD is in agreement with a few previous studies. Chen *et al*.^5^ reported that daytime napping duration of at least 30 min/d was associated with a 1.6-fold higher risk of osteoporosis in postmenopausal women^5^. Additionally, napping duration appeared to be detrimental to bone health in both pre- and postmenopausal women^9^. In a study of 4,962 postmenopausal women, daytime napping duration ≥30 min was associated with a 20–40% increase in the risk of osteopenia/osteoporosis; while in 1,548 premenopausal women, napping duration ≥60 min was also associated with 20–60% higher risk^9^. While there were slight differences in the methodology used for assessing BMD (DXA in our study and calcaneal QUS in the others), these results suggested a negative relationship between napping duration and bone health. The data in the current study further extended previous findings, as an association between napping duration and TH BMD was demonstrated in women with both normal BMD (31.3%) and osteopenia, while other studies mainly focused on women with osteopenia/osteoporosis.

Contrary to the findings at FN and TH, L2-4 BMD was not associated with napping patterns. It is possible that the association between napping/sleep pattern and BMD may differ by site (cortical vs. trabecular bone), although this has not been extensively examined. This presumption is supported by a study of 652 women in South Dakota, which revealed that only cortical, not trabecular, volumetric BMD was lower in sleep-deficient women (<6.5 h/night) when compared to those sleeping 6.5–10 h/night^12^.

There was no association between nighttime sleep duration and BMD in the current study, unlike findings in previous studies. A study of 2,693 postmenopausal women by Chen *et al*.^5^ reported that postmenopausal women with sleep duration of 7–8, 9–10 and >10 h/night had a risk of osteoporosis 1.5, 1.3 and 1.5 times greater, respectively, compared to those with sleep duration of 8-9 h/night^5^. Sex and ethnicity may influence this association, as longer nighttime sleep duration was found to be associated with lower FN BMD in Caucasian men, but not in women^13^, while the results were vice versa in the Korean population^14^. Lack of association between nighttime sleep duration and BMD in our study could also be due to differences in the distribution of sleep duration among the study populations, as the majority of our participants (44%) had nighttime sleep duration <7 h, while another study reported that the majority slept 8-9 h/night^5^

The mechanisms underlying the association between napping and bone health have not been extensively explored; however, there are several possible explanations. First, alterations in melatonin levels and circadian rhythm could play an important role. Melatonin, a neurohormone released from the pineal gland during the biological night, plays a significant role in human circadian regulation. Melatonin has been shown to promote osteoblast differentiation^15^ and suppress osteoclast differentiation^16,17^. Some studies in humans demonstrated that daytime naps were associated with alterations in melatonin secretion^18^. The effects of a 6 h daytime nap in darkness on melatonin secretion were explored in 25 healthy young men^18^. Compared to subjects who had no nap, a greater delay in melatonin onset was revealed in subjects who had a 6 h nap initiated in the morning, and a greater phase advance in melatonin onset was revealed in those who had a 6 h nap initiated in the evening^18^. In another study, elderly subjects who took evening naps had a more advanced acrophase (the time at which the peak of rhythm occurs) of 6-sulfatoxymelatonin, a major melatonin metabolite, when compared with those who did not nap^19^. Secondly, alterations in cortisol regulation could play a role in the association between napping and bone health^20^. Elderly individuals residing in a nursing home who had long napping duration (33.3 h/5 days) were found to have higher evening cortisol levels than those with short napping duration (13.6 h/5 days)^20^. Cortisol is known to disrupt bone formation and increase the risk of bone loss, and is associated with vertebral fractures^21,22^. Thirdly, insulin-like growth factor 1 (IGF-1), known to reduce osteoblast apoptosis and promote osteoblastogenesis^23^, possibly plays a role. Excessive daytime sleepiness, frequently reported in elderly patients who habitually napped^24^, was found to be related to lower IGF-1 levels^25^. However, there has been no study to date that directly examines the association between naps and IGF-1 levels in humans. Fourthly, inflammatory cytokines may additionally explain the relationship between bone health and napping pattern. A few studies in adolescents and adults found that napping was associated with higher interleukin 6 (IL-6) levels^26^ and higher high-sensitivity C-reactive protein (hsCRP) levels^27,28^. IL-6 has been shown to promote osteoclast differentiation and activation^29^, while hsCRP was found to be higher in osteopenic and osteoporotic women than in those with normal BMD^30^. Lastly, it is known that high napping frequency is associated with sedentary behavior or low physical activity in the elderly^31^. Lack of regular physical activity is a risk factor for bone loss in postmenopausal women^32^.

This study has the strength of being the first to explore the association between daytime napping characteristics and FN and TH BMD in elderly women. However, there are limitations. The cross-sectional design precluded assumption of a causal relationship. Information collected from volunteers may differ from population-based study and could be subjected to bias. Also, the sample size is small, thus possibly limited information on the exposure. Data regarding the intensity and duration of exercise were lacking, as well as information on melatonin, IGF-1 and inflammatory cytokine levels. Sleep characteristics were self-reported and not objectively measured. Other sleep parameters, such as snoring, are lacking. Lastly, the mechanisms underlying the association between daytime napping characteristics and BMD were not explored.

## Conclusion

This study demonstrated an association between daytime napping characteristics and BMD in elderly women. Higher daytime napping frequency and duration was associated with lower BMD, especially at FN and TH. Larger prospective studies are required to support the present findings.

## Methods

This study utilized the data collected from a previous study exploring the efficacy of calcium supplementation in elderly Thai women^33^, conducted during April 2002–April 2003. Three hundred and eighty-seven women were included in the study. The eligibility criteria were healthy volunteers aged 60 or older who had not taken any antiresorptive agents or estrogen within 6 months prior to enrollment. All subjects had a T-score > −2.5, as assessed by standard measurements with dual-energy X-ray absorptiometry (DXA). Exclusion criteria were major illnesses (metastatic metabolic disease), identifiable secondary causes of osteoporosis (glucocorticoid excess, hyperthyroidism or hyperparathyroidism), or consumption of calcium or vitamin D supplements within two months prior to enrollment. The protocol was approved by the Institutional Review Board, Faculty of Medicine Ramathibodi Hospital, Mahidol University, Bangkok, Thailand. All participants gave written informed consent. All methods were performed in accordance with relevant guidelines and regulations.

Subjects’ body weight and height were measured while wearing light clothing. Body mass index (BMI) was calculated as weight in kilograms divided by height in meters squared. Demographic data, including age, age at menopause, smoking, alcohol consumption and exercise habits, were obtained by interviews. Smoking status was reported as currently smoking vs. no. Alcohol consumption was defined by participants and reported as regular alcohol consumption vs. no. Similarly, exercise was defined by participants and reported as regular exercise vs. no. Medical history was extracted from medical records. T2DM was documented in participants who had a known history of diabetes or had been receiving antidiabetic drugs.

### Sleep Assessment

Nighttime sleep duration was derived from the question: “During the past year, how many hours of sleep did you get per night?” Additionally, napping was assessed using the following questions: “In the past year, did you typically nap?” and “If yes, how many times per week, and how many minutes each time?”

### Dietary Calcium Assessment

A three-day food record was obtained on each subject. All food records were analyzed for nutrient intake using INMUCAL software, which was developed by the Institute of Nutrition, Mahidol University, Bangkok, Thailand^34^.

### Vitamin D Assessment

Sera were collected and stored at −80 °C until assayed; all samples were processed together in one batch at the end of the study 12. Serum 25-hydroxyvitamin D [25(OH)D] was measured by radioimmunoassay (DiaSorin Inc., Stillwater, MN) with an intra-assay precision of 8.9%.

### Bone Mineral Density Assessment

Each subject changed into light clothing before undergoing BMD assessment by DXA at the lumbar spine (L2–L4 vertebrae) and hip [femoral neck (FN) and total hip (TH)]. Using fast-array mode, all measurement procedures were performed according to the International Society for Clinical Densitometry (ISCD) recommendations 13 by a single experienced technician using the same DXA scanner (Lunar PRODIGY™; GE Healthcare, Madison, WI, USA) on all subjects. Quality control was done by daily calibration and phantom scans. The coefficient of variation for phantom scans was 0.6% *in vivo*, and these values were 1.2%, 1.6% and 1.3% at L2-4, FN and TH, respectively. According to World Health Organization criteria, BMD T-score ≥ −1 at all sites (L2-4, FN and TH) was defined as normal, and BMD T-score between −1 and −2.5 at any site (L2-4, FN or TH) was defined as osteopenia^35^.

### Statistical Analysis

Data are presented as mean^10^ or percentage. Univariate linear regression analyses were performed to explore the associations between BMD and demographic and sleep variables. A hierarchical multiple regression analysis was used to determine the independent predictors of BMD. Demographics, lifestyle, dietary calcium intake and 25(OH)D associated with L2-4, FN or TH BMD in the univariate analyses (with p < 0.1) were entered in the first step. In the second step, sleep variables were entered. There was no collinearity among the variables. SPSS version 18.0 (Chicago, IL) was used to analyze the data. A p-value < 0.05 was considered statistically significant.
